# Exercise and cognitive function: a hypothesis for the association of type II diabetes mellitus and Alzheimer's disease from an evolutionary perspective

**DOI:** 10.1186/1758-5996-1-7

**Published:** 2009-09-18

**Authors:** Gilberto NO Brito

**Affiliations:** 1 Setor de Neurociências, Departamento de Pediatria, Instituto Fernandes Figueira, FIOCRUZ, Rio de Janeiro, RJ, Brasil; 2 Departamento de Psiquiatria e Saúde Mental, Instituto de Saúde da Comunidade, Universidade Federal Fluminense, Niterói, RJ, Brasil

## Abstract

The association of type II diabetes mellitus (DM2) with Alzheimer's disease (AD) has received considerable attention in recent years. In the present paper, a hypothesis for this association from an evolutionary perspective, with emphasis on the close interplay between exercise and cognitive function, will be advanced in order to provide a biological rationale for the notion that the fundamental metabolic features of DM2 act in the brain over a protracted time span to induce the neuropathological characteristics of Alzheimer's disease thereby producing cognitive impairment. It is hoped that this hypothesis puts the association of DM2 and AD on firm conceptual grounds from a biological perspective and offers directions for further research.

## 

The great evolutionary biologist Theodosius Dobzhansky once said: "Nothing in biology makes sense except in the light of evolution". Along this line, I will put forward a hypothesis for the association of type II diabetes mellitus (DM2) and Alzheimer disease (AD) from an evolutionary perspective. This hypothesis contends that the close interplay between physical activity/exercise and cognitive function was selected for during the course of evolution, but the absence of sufficient physical activity typical of modern societies provoked a rupture of the evolutionary relationship of exercise and cognitive function thereby producing the fundamental metabolic features of DM2 which in the brain over a protracted time span lead to the neuropathology of AD. I will begin by posing the issue of the association of DM2 and AD and then proceed with a brief review of the endurance running hypothesis for the evolution of man. Next, the neurobiological and neurobehavioral effects of exercise/physical activity will be discussed and then I will present the hypothesis. Lastly, directions for further research on the basis of the hypothesis will be suggested.

Although both physical activity and exercise involve bodily movement produced by skeletal muscles, exercise, but not physical activity, incorporates a goal for the physical activity namely to improve or maintain physical fitness. Be that as it may, in the present paper physical activity and exercise will be used interchangeably.

## The issue

DM2 and AD are common in the elderly population the world over. Since these two disorders are both chronic and progressive, their combined economic burden on society has skyrocketed *pari passu *with the aging of the population. Therefore, DM2 and AD have become two of the most important diseases in the geriatric health care practice.

The prevalence of DM2 in people older than 65 years in Western societies is 8.5% [1] and the corresponding figure for AD is about 30.0% at 80 years [2]. Thus, the prevalence rates of DM2 and AD would make the association of the two disorders likely on statistical grounds alone, i.e., as the average age of the population advances, the prevalence of DM2 and AD increases and so does the probability of association of the two disorders. However, there seems to be a consensus among researchers in the field that DM2 increases the risk of AD above the expected probability of the association of the two disorders with aging [3,4].

**Figure 1:**
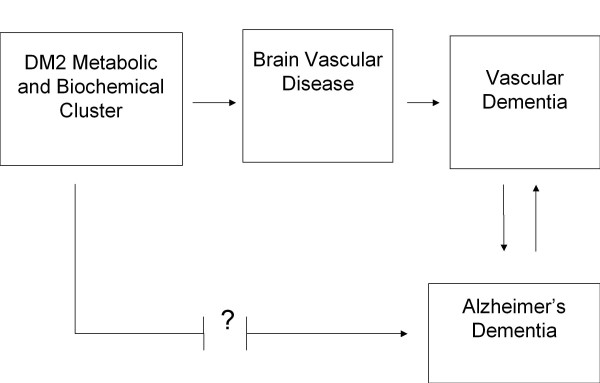
**A diagrammatic illustration of the possible pathways for the association of DM2 and dementia**.

The relevant issue for research becomes whether DM2 is specifically associated with the neural substrate of AD independently of the well-known effects of the metabolic and biochemical cluster associated with diabetes on the brain vasculature (see Fig. 1). Nevertheless, even if DM2 is not specifically associated with the neural substrate of AD, DM2-induced microvascular injury in the brain could trigger the appearance of the neuropathological hallmarks of AD over time, or else, accentuate incipient AD-related neuropathology. In this regard, a recent report pointed out that the pathological line between vascular dementia and AD is not at all clearcut [5].

## The endurance running hypothesis for the evolution of man

A major function of the brain is to mediate behavioral expression and therefore to coordinate the action of cross-striated or skeletal muscle fibers. In our species, about 40% of body weight is composed of skeletal muscle, which makes it the largest tissue type in the body. In addition, it has been estimated that skeletal muscle uses up to 25% of the energy consumed by the body at rest. This extraordinary power plant evolved over time to provide our ancestors with sophisticated metabolic capabilities to sustain high levels of physical activity and thus become successful hunter-gatherers.

Although humans are not considered particularly good sprinters, they can engage in a type of long-distance running over extended time periods using aerobic metabolism, also known as endurance running. Endurance running seems to be a unique human feature among primates and is uncommon in quadrupedal mammals with the exception of some social carnivores such as hyenas and dogs and migratory ungulates such as horses and wildebeests [6]. A study recently published [7] presented evidence which supports a biomechanical model of toe function in bipedal locomotion consistent with the endurance running capabilities of humans. Our short toes allow us to run considerable distances very effectively in terms of metabolic cost. In spite of limitations in the fossil record, it seems that the capability of endurance running in hominids first appeared in *Homo erectus *about two million years into the past perhaps for the purpose of hunting since it would make it possible for our ancestors to get close enough to throw projectiles or possibly run four-legged prey to heat exhaustion, the so-called persistence hunting [6]. At the present time, endurance running is seen as exotic and practiced only by ultra-distance runners, but it has been conserved in cultures like the Tarahumara of Mexico [8]. Interestingly, the Tarahumarans seem to be immune to cancer and other diseases common in industrialized societies such as DM2.

## Exercise, neuroplasticity and cognitive function

It is likely that the endurance, and for that matter locomotor, capabilities of early humans also permitted that they built detailed cognitive maps of their environments as they ran and walked along in order to locate scavenging opportunities, possible prey and escape routes from predators. Therefore, it could be envisaged that physical activity and cognitive function evolved in a very close fashion.

Research in experimental animals has shown that the hippocampal system has a major role in cognitive function [9] which seems to be mediated by cholinergic projections from the basal forebrain [10]. Interestingly, insulin signalling-related proteins coexist with choline acetyltransferase in terminal buttons located in CA1 hippocampal pyramidal cells [11] and this finding raises the possibility that the insulin and the cholinergic systems of the hippocampus interact in the mediation of cognitive function.

It is therefore of significant relevance that experimental studies have demonstrated that physical exercise induces neurobiological changes in the very same brain systems involved in cognitive function. Physical exercise increases mRNA levels of BDNF (brain-derived neurotrophic factor) and NGF (nerve growth factor) [12,13], transcripts of neuropeptide-related genes [13], genes coding for extracellular matrix proteins and biosynthetic processes [13] in the hippocampus. Furthermore, it has been shown that exercise increases small heat shock proteins (sHSP) and pre- and postsynaptic proteins [14], synapsin and synaptophysin [15] in the hippocampus and such proteins are known to play a role in synaptic plasticity. Moreover, the selective blockade of the BDNF receptor abolishes the exercise-induced elevations of synapsin and synaptophysin in the hippocampus showing that exercise modulates synaptic properties under the direction of BDNF [15].

The neurobiological changes induced by physical exercise in the hippocampus have been demonstrated to facilitate the acquisition of a spatial memory task in rats, the radial arm maze [16]. Additionally, rats that are fast learners in a similar spatial-memory task (water maze) showed the highest expression of BDNF and associated cAMP response-element-binding protein (CREB) and synapsin in the hippocampus [17]; and blockade of the BDNF receptor reduced the cognitive capabilities of rats to sedentary levels [17]. Moreover, a neurophysiological study showed that the increased gene-expression of BDNF correlated with enhancement of synaptic transmission within a population of hippocampal pyramidal cells [18] and BDNF is known to induce long-term potentiation, a type of synaptic plasticity related to learning and memory, in the hippocampus [19]. Furthermore, sedentary rats show reduced brain uptake of serum insulin-like growth factor I (IGF-I) compared to animals that exercise and IGF-I uptake is important for exercise-induced neurogenesis in the hippocampus [20] and, additionally, IGF-I improves the memory performance of aged rats [21]. Interestingly, it has been recently shown that exercise prevents deficits in spatial memory of streptozotocin-induced diabetic rats [22].

Human studies also demonstrate that physical activity and exercise exert a significant impact on cognitive function in preadolescent children [23] and older adults [24]. Moreover, a home-based program of physical activity provides an improvement in cognition in adults with subjective memory impairment [25] and regular exercise is associated with reduced risk for incident dementia in the elderly [26]. Furthermore, aerobic fitness promotes faster processing of stimulus encoding, facilitates executive control of behavior, increases brain volume, induces changes in activation patterns in functional brain MRI and increases measures of cerebral blood volume in the hippocampus of middle-aged adults [see [27] for a review].

Exercise induces plastic changes not only in the brain but also in the skeletal muscle. Lack of physical activity can make a substantial part of skeletal muscle tissue disappear and, conversely, a physical training program forces the muscle to remodel itself to allow energy to be used more efficiently when it contracts. It has been demonstrated that the activity level of skeletal muscle modulates gene expression in such a way as to facilitate, for example, the effect of insulin in moving glucose into the muscle [28]. In a resting muscle cell, the glucose transporter protein GLUT4 is located in vesicles below the plasma membrane and in response to insulin or exercise these GLUT4 vesicles fuse with the plasma membrane to transport glucose from the blood into the cell; a regular exercise program induces an increase in the production of GLUT4 protein through activation of its gene and physical inactivity decreases GLUT4 to levels found in patients with DM2 [28]. It is likely that similar changes in GLUT4 activity also occur in the brain. Along this line, it has been reported that the combination of aerobic and resistance training provides the greatest improvement in glycemic control in patients with DM2 than either type of training alone [29].

It can be concluded that exercise and physical activity are associated with tandem dynamic and plastic changes in brain and skeletal muscle which impact significantly on cognitive function.

## A hypothesis for the association of DM2 and AD from an evolutionary perspective

The evidence reviewed above highlights that physical activity has a significant impact on cognitive function with the probable mediation of insulin-related systems in the brain and skeletal muscle. It can be surmised that physical activity permits the construction of detailed cognitive maps of the environment as the organism moves around its surroundings. Map-building capabilities associated with physical activity allow for the location of resources such as food and also escape routes from predators. Therefore, the association of physical activity and cognition provided the organism with a survival advantage and was probably selected for over the course of evolution.

Natural selection states that genetic variants of a population will be more successful the more copies of their genes are put into the next generation. Since very few individuals in the wild will live to old age due to high mortality rates for a variety of reasons, including predation and illness, the great majority of the wild population consists mostly of young adults who will make the genetic contributions to the next generation. It follows that deleterious genetic variants that act late in life will not be selected against because their carriers will not live long enough to contribute their genes to the gene pool of the population or will survive as post-reproductive adults. In any case, their deleterious genes would be imperceptible to natural selection because these genes would be expressed after reproduction and natural selection acts strictly on reproductive success. Along this line, reaching old age per se has no evolutionary merit unless it comes along with a significant contribution to the genetic pool of the population. Simply stated, if you die in senescence but leave no offspring, your genes will not contribute to the genetic pool and you become an evolutionary dead-end. It should be granted, however, that, senescence provides opportunities for altruistic behavior in the form of intergenerational help as, for example, in raising the young. Such altruistic behavior among relatives may increase the likelihood of maintaining one's genes in the genetic pool of the population in the absence of offspring. Be that as it may, senescence per se is by and large imperceptible to natural selection on an individual basis.

As recently as 1900, humans lived about 40 to 45 years, whereas at the turn of the third millennium they lived 80 years certainly due to efficient public health and medical interventions developed during the last century. These 40 years of extended life expectancy are entirely post-reproductive and, therefore, invisible to natural selection, as mentioned above. Studies in the biology of aging have shown that extreme stability in gene expression can lead to a centenarian life and, conversely, unstable expression may lead to premature mortality and some biomarkers such as insulin levels are associated in an inverse fashion with mortality [30]. The reason that we are not capable of living and reproducing *ad eternum *seems to be related to limits in the allocation of energy to reproduction and maintenance/repair of the cellular machinery; more allocation of energy to reproduction means less to maintenance/repair and so to decreased survivorship and vice-versa [30]. However, it should be emphasized that the extreme allocation of energy to maintenance/repair to the detriment of reproduction will lead to the organism not contributing genes to the gene pool of the population, essentially becoming an evolutionary dead-end.

Advances in medical and social interventions and more recently also in technology have made our lives, not only longer, but also more fulfilling than at any other time in our evolutionary history. We no longer have to hunt to gather food and the availability of remotes makes us comfortable in our sofas to watch our favourite TV programs at the simple movement of a finger! How sedentary have we become! Herein lies the greatest challenge in our pursuit of longer and happier lives. How to maintain our evolutionary conquests in light of the facilities afforded by our modern societies?

We evolved a sophisticated fabric of skeletal muscle and neurons which working in harmony allowed us to produce cognitive behavioral patterns that permitted the passing of our genes through the ages thus making our species very successful in the evolutionary game. It is this very same fabric that is being now thrown into the waste basket. Lack of physical activity so typical of our modern society has provoked a rupture in the delicate metabolic harmony of skeletal muscle and neural function that has served us so well to provide our superb cognitive capabilities and thus our success as a species. It is this rupture in the evolutionary relationship between skeletal muscle function in the form of exercise and neural function in the form of cognition that is behind the natural fracture line induced in our behavioral capabilities in the form of cognitive impairment which appear ever more frequently in our post-reproductive lives.

It is therefore plausible to propose the hypothesis that the reported association between DM2 and AD [3,4] in fact represents the clinical correlate of the natural fracture line in our behavioral capabilities provoked by the rupture of the evolutionary relationship between skeletal muscle and neural functions which leads over our post-reproductive years to the neuropathology of AD. In this sense, the neuropathological features of AD found in patients with DM2 independently of the presence of microvascular injury may reflect a disruption in brain's insulin-related systems in the form of a diminished sensitivity of insulin receptors or accumulation of advanced glycation end products related to chronic intermittent hyperglycemia [31]. Along these lines, a recent report showed that insulin resistance in the brain is associated with a decrease in theta activity with aging [32] and theta activity is important for hippocampus-dependent cognitive function [33]. It is likely that the natural fracture line referred to above includes the hippocampal system. Therefore, it can be hypothesized that changes in the function of the insulin systems of the brain possibly induced by the rupture of the evolutionary relationship between skeletal muscle and neural functions would be entirely consistent with the notion that these changes represent type III diabetes mellitus (DM3) as suggested by some investigators [34].

It is hoped that the hypothesis proposed above has heuristic value in the quest for the understanding of the association of DM2 and AD at least in the sense that it puts the association of these two devastating disorders on a firm conceptual footing from a biological perspective. Furthermore, this hypothesis has important implications for preventative medicine and clinical and experimental research, as we shall comment below.

## Implications of the hypothesis for the clinic and research

The hypothesis presented in this paper has significant implications for efforts directed to the prevention of the metabolic consequences of a sedentary lifestyle from early on in development. Public health should combat forcefully the obesity epidemics in children, adolescents and adults. Physical activity should be encouraged in school curricula as if it were a major discipline not only because of its effects on metabolism but also due to its impact on academic achievement as discussed previously in this paper. Moreover, physical activity should also be encouraged in every member of each community in order to maintain the harmony between skeletal muscle and brain function and thereby prevent cognitive impairment late in life. In fact, the recommendation for exercise since early on in development is in complete agreement with the idea that to enrich the environment is to empower the brain [35] and such empowerment has profound implications for recovery of function after neural insult. In addition, as the brain is empowered the brain reserve capacity is boosted which is important for the prevention of neurodegenerative disorders.

Clinical investigation should scrutinize closely the effects of dynamic alterations in glycemia during development on neurobehavioral function in order to ascertain the neurotoxicity of glucose [36] early on and verify the effects of intervention on cognitive function. Glucose levels and glucose tolerance might be relevant parameters of the degree of metabolic stress on insulin-related systems and the examination of their validity in regard to neurocognitive function may provide important clinical information as recent evidence appears to indicate [37,38]. Brain imaging studies [39] could also be used to evaluate the neurobiological validity of glucose levels as an index of the metabolic stress on the insulin systems of the brain. In addition, clinical-pathologic studies in the neuropathology of DM2 with proper control of the many possible confounding variables [40] are in much need in order to establish possible specific brain correlates of DM2.

Research in experimental animals could provide model systems for the study of the deleterious effects of hyperglycemia or hyperinsulinemia on the brain vasculature and neuronal chemo-architecture with AD transgenic animals [41]. Experimental studies could include a protocol for neurobehavioral assessment to establish effects on cognitive function. Furthermore, the effects of exercise on cognitive function in these model systems could be evaluated as regards, for example, the possibility of interaction between exercise and dose of antidiabetic drugs. Moreover, additional experimental studies on the complexity of the interaction between insulin-signalling systems and amyloid peptides [42] are needed to open up new possibilities for the treatment of AD and the role of antidiabetic drugs such as metformin in the modulation of the metabolism of amyloid precursor protein [43] should be clarified in order to delineate its potential harmful effects in DM2 patients.

It is expected that such research provides conclusive evidence for the still tentative association of DM2 and AD and it is hoped that the hypothesis herein presented offers a conceptual biological basis for the continuation of this endeavor.

## Competing interests

The author declares that he has no competing interests.

## Authors' contributions

GNOB conceived the study and prepared the manuscript.
